# Role of Standardized Plant Extracts in Controlling Alcohol Withdrawal Syndrome—An Experimental Study

**DOI:** 10.3390/brainsci11070919

**Published:** 2021-07-12

**Authors:** Ijasul M. Haque, Akhilesh Mishra, Bhupinder Singh Kalra, Shalini Chawla

**Affiliations:** 1 Department of Pharmacology, Maulana Azad Medical College, New Delhi 110002, India; ijasulhaqmm@gmail.com (I.M.H.); drbskalra@gmail.com (B.S.K.); 2 Central Animal Facility, Maulana Azad Medical College, New Delhi 110002, India; drakhileshvet@gmail.com

**Keywords:** Ashwagandha, Brahmi, alcohol withdrawal syndrome, seizures, diazepam

## Abstract

Patients with alcohol use disorder experience alcohol withdrawal syndrome due to the sudden cessation of alcohol. This study was designed to evaluate the protective effect of Ashwagandha and Brahmi on alcohol withdrawal in rats. Thirty rats of either sex were taken and randomly divided into 6 groups (*n* = 5). Their normal diet was replaced by a modified liquid diet (MLD). Ethanol was added gradually except in the MLD group for a period of 21 days and withdrawn suddenly. Four treatment groups were administered Ashwagandha (3.75 mg of withanolide glycosides per kg body weight), Brahmi (10 mg of bacosides per kg body weight), Ashwagandha + Brahmi (3.75 mg withanolide glycosides + 10 mg bacosides per kg body weight) orally and diazepam (1 mg/kg body weight, i.p.) 45 min before alcohol withdrawal. Rats were assessed for behavioural changes (agitation score and stereotypic behaviour), anxiety and locomotor activity at 2nd and 6th hours of alcohol withdrawal. Pentylenetetrazol (PTZ) kindling seizures were assessed at 6th hour of alcohol withdrawal. Ashwagandha and Brahmi alone and in combination significantly reduced the behavioural changes in alcohol withdrawal rats at 2nd hour and their combination in 6th hour. Ashwagandha and Brahmi suppressed PTZ kindling seizures effectively and improved locomotory activity at 2nd hour and 6th hour of alcohol withdrawal. Reduction in anxiety was significant among Ashwagandha at 2nd hour and the combination group at 2nd and 6th hour. The results were comparable to diazepam. Ashwagandha and Brahmi have beneficial effects in controlling the behavioural changes, anxiety and seizures in alcohol withdrawal symptoms in rats and improved locomotory activity.

## 1. Introduction

Alcohol (ethanol or ethyl alcohol) is a commonly used psychoactive substance with dependence-producing properties. Globally, alcohol use disorder (AUD) is a major health problem and is associated with social, mental, physical and legal consequences. It is generally associated with cravings, tolerance and withdrawal symptoms upon cessation [1]. Alcohol withdrawal syndrome (AWS) is a well-known condition occurring after intentional or unintentional abrupt cessation of heavy or chronic drinking and it occurs in about 8% of hospitalized AUD inpatients [2]. AWS is characterized by tremors, anxiety, sweating, nausea and tachycardia and in severe cases, may involve hallucinations, seizures and delirium tremens. Untreated, AWS can be fatal [1].

Alcohol is a central nervous system depressant that produces euphoria and behavioural excitation at low blood concentrations due to increased glutamate binding to N-methyl-D-aspartate (NMDA) receptors; at higher concentrations, it leads to acute intoxication by potentiation of the GABA effects [3]. Prolonged alcohol use leads to the development of tolerance and physical dependence, which may result from compensatory functional changes by downregulation of GABA receptors and increased expression of NMDA receptors. Abrupt cessation of chronic alcohol consumption unmasks these changes with a glutamate-mediated CNS excitation resulting in autonomic overactivity and neuropsychiatric complications such as delirium and seizures [4]. Thus, patients experiencing alcohol withdrawal have decreased GABA-ergic functioning and increased glutamatergic action throughout the central nervous system.

Management for any patient with suspected alcohol withdrawal is initial resuscitation and rehydration. Benzodiazepines (BZD) are the first line pharmacological agent for treating AWS. The benzodiazepines most used for this purpose are lorazepam, chlordiazepoxide, oxazepam and diazepam. They act as central GABA-A agonists, increasing the frequency of GABA-receptor channel opening leading to more influx of chloride ions and providing an inhibitory effect which is similar to that of ethanol [5]. BZDs are associated with potentially life-threatening side effects, including the risk of dependency and withdrawal symptoms, increased risks of falls and respiratory failure. In addition, psychological side effects, cognitive impairments, depressive mood and sleep disorders have been described, as well as increased impulsivity that could lead to suicidal behaviours [6]. In view of the available literature, there is a need for better and safer pharmacological agents for managing AWS.

*Withania somnifera*, commonly known as Ashwagandha, has been used for centuries in Ayurvedic medicine to increase longevity and vitality [7]. Various preclinical studies have shown that Ashwagandha has anticonvulsant [8], antidepressant [9], antianxiety [10], hepatoprotective [11] effects, etc. Ashwagandha preparations have been found to have a potential therapeutic role in almost every CNS-related disorders. Glycowithanolides withaferin- A and sitoindosides VII–X isolated from Ashwagandha significantly reversed ibotenic acid-induced cognitive defects in Alzheimer’s disease model [12]. Another study demonstrated that chronic oral administration of withanoside attenuated the axonal, dendritic and synaptic losses and memory deficits induced by amyloid peptide Aβ (25–35) in mice [13]. It can modulate GABAergic, cholinergic and oxidative systems in CNS [14].

*Bacopa monnieri*, commonly known as Brahmi is a nootropic plant in Ayurveda that has been studied widely for its cognition-enhancing, antidepressant, antihypertensive, anti-asthmatic, antiulcer, analgesic, neuroprotective, hepatoprotective and nephroprotective properties. Bacoside-A is the major chemical entity responsible for Brahmi’s well-known nootropic effect as well as other neuromodulatory, hepatoprotective and, antioxidant activities [15]. Few preclinical studies have shown that Brahmi treatment potentiates a therapeutic effect by reversing the alterations in GABA and GABA_A_ receptor binding that occur during epilepsy, resulting an increased GABA mediated inhibition in the overstimulated cerebral cortex neurons [15,16]. Another study showed improvement in spatial learning performance and enhanced memory retention in rats treated with Brahmi extract clearly indicating that exposure to Brahmi improved learning and memory [17]. Brahmi also showed strong and dose-dependent antidepressant effects in different mice models [18]. Another study in epileptic rat model concluded that Brahmi potentiates a therapeutic effect by reversing the alterations in general GABA, GABAA, GABAB receptor binding, GABAA receptor subunits, GAD and CREB gene expression that occurs during epilepsy, resulting an increased GABA mediated inhibition in the over stimulated cerebral cortex neurons [16].

In preclinical studies, animals exhibit alcohol abstinence symptoms like those observed in humans. The anxiety and depressive-like behaviour in rodent models mimics human dysphoric emotion related to alcohol abstinence and may aid in testing the therapeutic potential of promising agents for alcoholism [19]. In most of the studies, modified liquid diet model of chronic ethanol administration was used. A study was done to validate the modified liquid diet model of chronic ethanol administration. They concluded that it may be used as a valid means to investigate ethanol abuse and dependence in an experimental context and can be used to assess the pharmacological profile of drugs on alcohol withdrawal syndrome in rats [20].

Ashwagandha and Brahmi with their GABA agonist activity may have the potential to control alcohol withdrawal symptoms. The objective of the study was to study and compare the effect of *Withania somnifera* (Ashwagandha) and *Bacopa monnieri* (Brahmi) individually and their combination with Diazepam on alcohol withdrawal syndrome in rats.

## 2. Materials and Methods

### 2.1. Materials and Reagents

Ashwagandha (*Withania somnifera*) extract containing 35% withanolide glycosides and Brahmi (*Bacopa monnieri*) extract containing 21.4% total bacoside were supplied by Arjuna Natural Pvt. Ltd., Aluva, India. Diazepam and ethanol were purchased and used in the study. Reference standards of Withaferin A (% purity ≥ 99.0), Withanoside IV (% purity ≥ 99.0) and Withanoside V (% purity ≥ 99.0) were purchased from Chromadex (Los Angeles, CA, USA) and Bacoside A3 (% purity ≥ 99.0) were purchased from Sigma. Acetonitrile, methanol and water (LCMS grade) were purchased from Merck (Mumbai, India). The Waters^®^ ACQUITY UPLC (Milford, MA, USA) with a photo-diode array (PDA) detector and Triple Quadrupole (Waters Quattro Premier XE, Poway, CA, USA) mass spectrometer with an electrospray ionization (ESI) source was used for the mass spectrometric analysis.

### 2.2. LCMS Analysis Method of Ashwagandha Extract

**Figure 1:**
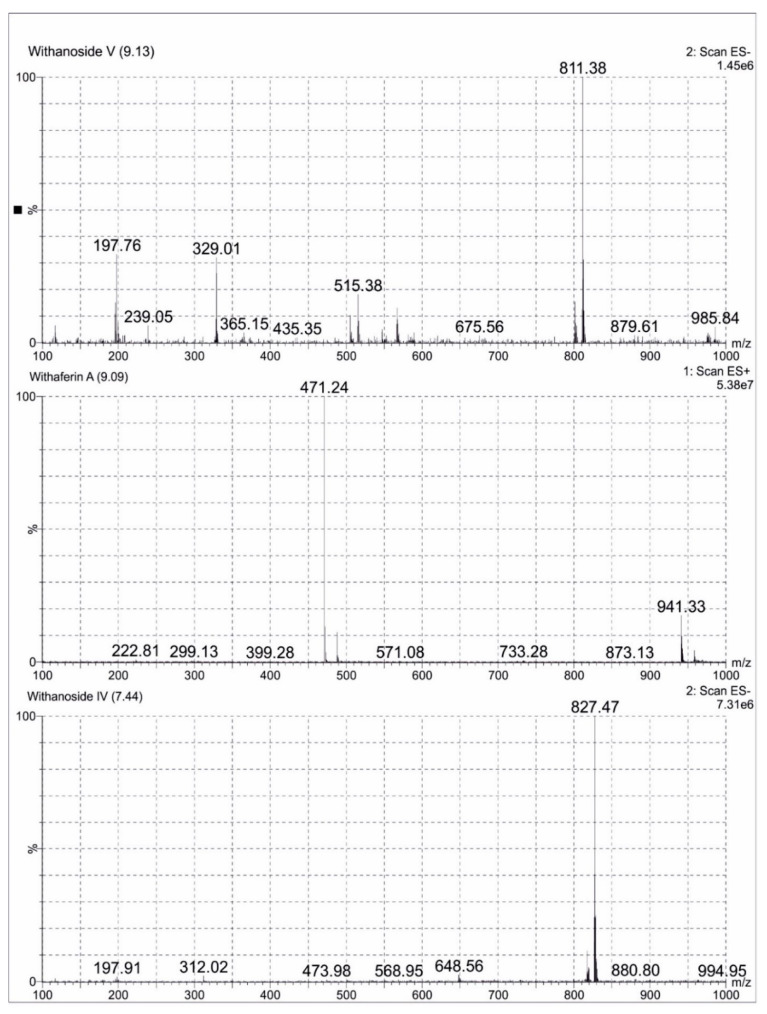
Negative and Positive ion electrospray ionisation liquid chromatography -mass spectrometry mass spectrum (ESI-MS) of Withanoside V, Withaferin A and Withanoside IV separated in Ashwagandha extract.

A total of 0.1 mg/mL of reference standards Withaferin A, Withanoside IV and Withanoside V diluted in 25 mL methanol (LCMS grade) were prepared. A total of 10 mg of Ashwagandha extract was dissolved in methanol (LCMS grade) and made up to 25 mL volumetric flask. The sample was clarified using Millipore filters (0.22 µm), degassed for one minute and subjected to LCMS analysis. Analytes were quantified using MRM method. Withanoside IV and Withanoside V were ionized in the negative mode and Withaferin A was ionized in the positive mode. All the MS parameters were manually fine-tuned to obtain the highest MRM signals. The following MRM transitions of precursor ions to product ions were used: m/z 827.441→763.359 for Withanoside IV; m/z 811.063→765.51 for Withanoside V; and m/z 471.171→94.95 for Withaferin A. The compounds were separated on a C18 Kinetex column (50 mm × 2.1 mm × 1.7 μm) at a column temperature of 35 °C. The mobile phase was composed of water (A) and acetonitrile (B) with a gradient elution program as per Aboli Girme et al. The chromatograms were acquired at a flow rate of 0.3 mL/min with an injection volume of 5 μL [21]. ESI-MS spectra of Ashwagandha extract showing Withanoside IV at Rt 7.44 min m/z 827.47 formate adduct, Withaferin A at Rt 9.09 min m/z 471.29 (M + H)^+^ and Withanoside V at Rt 9.13 min m/z 811.38 formate adduct were represented in Figure 1.

### 2.3. LCMS Analysis Method of Bacopa Extract

**Figure 2:**
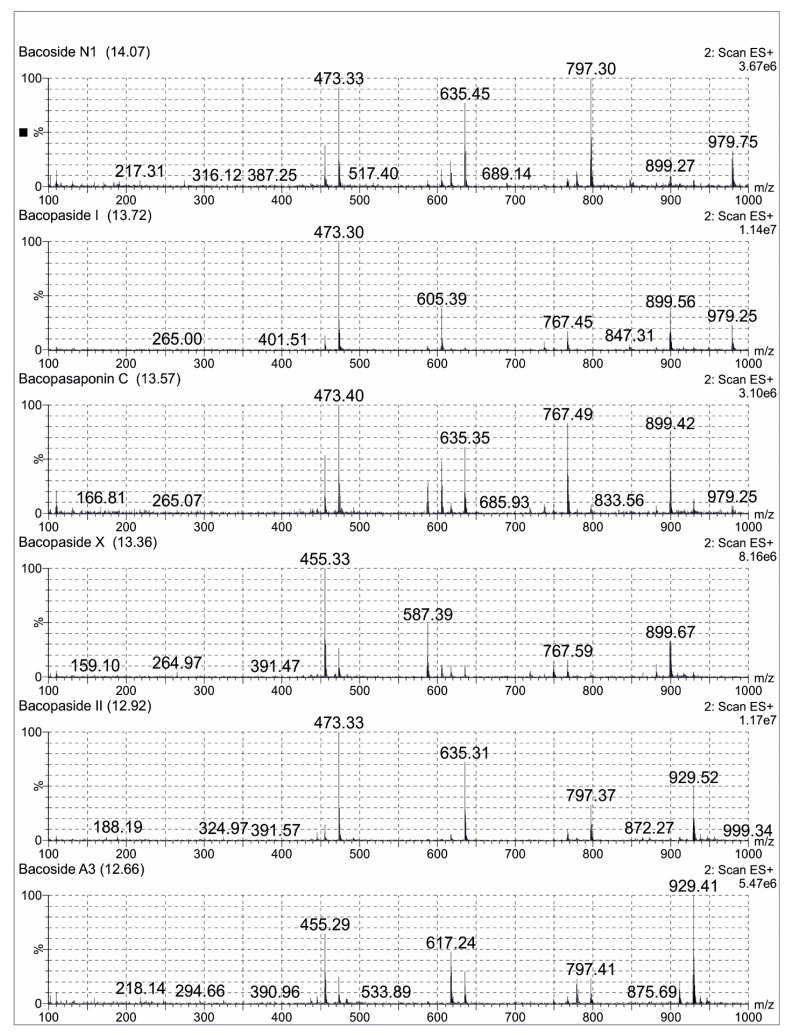
Positive ion electrospray ionisation liquid chromatography -mass spectrometry mass Scheme 1. Bacopaside 1, Bacopasaponin C, Bacopaside X, Bacopaside II and Bacoside A3 separated in Brahmi extract.

A total of 0.1 mg/mL of the reference standard of Bacoside A3 diluted in 25 mL methanol (LCMS grade) was prepared. A total of 25 mg of Bacopa extract was dissolved in methanol (LCMS grade) and was made up to 50 mL volumetric flask. The sample was filtered using Millipore filters (0.22 µm) for one minute and subjected to LCMS analysis. An aliquot of 10 μL of the sample solution was used for LC-MS analysis with a total run time of 16 min. Mass spectra were recorded in the positive ionization mode and with the full scan (m/z 100–1000). The compounds were separated on a C18 Kinetex column (50 mm × 2.1 mm × 1.7 μm) with a column temperature of 45 °C. The mobile phase was composed of 0.1% formic acid in water (A) and 0.1% formic acid in acetonitrile (B) with a gradient elution program. The chromatograms were acquired at a flow rate of 0.3 mL/min. All the acquisition and analysis of the data were controlled by Mass Lynx Data Acquisition Software version 4.1. TIC of the samples exhibited several peaks along with their retention time (Rt) indicating the presence of bacosides in the Bacopa extract. The jujubogenin glycosides (Bacoside A3 and Bacopaside X) showed the characteristic m/z 455 [aglycone + H-H_2_O]^+^, while pseudojujubogenin glycosides (Bacopaside I, Bacopaside II and Bacopasaponin C) showed the main fragment ion at m/z 473 [aglycone + H]^+^. Some of the important possible bacosides were identified with the help of the ESI-MS spectral analysis, which were already reported [22]. The possible bacosides identified in sample were Bacoside A3 at Rt of 12.66 min, m/z 929. A total of 41 [M + H]^+^, Bacopaside II at Rt of 12.92 min, m/z 929.52 [M + H]^+^, Bacopaside X at Rt of 13.36 min, m/z 899.67 [M + H]^+^, Bacopasaponin C at Rt of 13.57 min, m/z 899.42 [M + H]^+^, Bacopaside I at Rt of 13.72 min, m/z 979.25 [M + H]^+^ and Bacoside N1 at Rt of 14.07 min m/z 767.41 [M + H]^+^ (Figure 2).

### 2.4. Animals and Treatment

Thirty Sprague Dawley rats of 6 to 8 months old, weighing 200–300 g of either sex was used for the study. During the study period, each rat was caged individually in a quiet room under ideal temperature and light. Their normal diet was replaced with a modified liquid diet (MLD) ad libitum [20]. This animal experimental study was conducted in the Department of Pharmacology, Maulana Azad Medical College, Delhi with the approval of the Institutional Animal Ethical Committee of Maulana Azad Medical College. The study was conducted within the framework of CPCSEA (Committee for purpose of control and supervision of experiments on animals) guidelines.

Rats were divided randomly into 6 groups with five animals in each group as follows: Group 1—Vehicle control group treated with Modified liquid diet (MLD) (Vehicle control group), Group 2—Modified Liquid diet (MLD) + Ethanol, Group 3—MLD + Ethanol + Diazepam (1 mg/kg IP), Group 4—MLD + Ethanol + Ashwagandha (3.75 mg of withanolide glycosides per kg body weight), Group 5—MLD + Ethanol + Brahmi (10 mg of bacosides per kg body weight), Group 6—MLD + Ethanol + Ashwagandha + Brahmi (3.75 mg of withanolide glycosides + 10 mg of bacosides per kg body weight).

### 2.5. Establishment of Alcohol Dependence

For chronic ethanol exposure, the rats were housed individually and ethanol was given in the modified liquid diet. No extra chow or water was supplied. The composition of the modified liquid diet with ethanol was cow milk 925 mL, 25–75 mL ethanol (96.5% ethyl alcohol), vitamin A 5000 IU and sucrose 17 g [20]. This mixture supplies 1000.7 kcal/L.

At the beginning of the study, all the rats were given the modified liquid diet without ethanol for 7 days. Then, a liquid diet with 2.4% *v/v* ethanol was administered for 3 days. The ethanol concentration was increased to 4.8% *v/v* for the following 4 days and finally, to 7.2% *v/v* for 14 days. A liquid diet was freshly prepared daily and presented at the same time of the day. The weight of the rats was recorded every day. Control rats were fed with an isocaloric liquid diet containing sucrose as a caloric substitute to ethanol. At the end of exposure to 7.2% *v/v* ethanol-containing diet, ethanol was withdrawn from the diet after 21 days of administration.

Diazepam (1 mg/kg body weight, I.P.), Ashwagandha (3.75 mg of withanolide glycosides per kg body weight, oral), Brahmi (10 mg of bacosides per kg bodyweight, oral) and Ashwagandha +Brahmi ((3.75 mg of withanolide glycosides + 10 mg of bacosides per kg body weight) were administered to the rats 45 min before ethanol withdrawal in the respective groups. Diazepam, Ashwagandha, Brahmi and Ashwagandha + Brahmi were continued once daily dose for 5 days.

### 2.6. Assessment

Behavioural changes, anxiety, locomotor activity and Pentylenetetrazole (PTZ) kindling seizure were assessed by the investigators who were blinded regarding the treatment given to the animals. The rats were observed for 5 min at second and sixth hours of the withdrawal period for agitation and stereotyped behaviour. After 2 h and 6 h of ethanol withdrawal, anxiety was assessed with an elevated plus maze (EPZ) and locomotor activity was assessed with an open field test (OFT). After 6 h of ethanol withdrawal, the effect of subconvulsive dose of PTZ was assessed.

#### 2.6.1. Agitation and Stereotyped Behaviour

Agitation is the state of irritability and aggressive behaviour. Grooming, sniffing, head weaving, gnawing and chewing were observed as major stereotyped behaviours during the ethanol withdrawal in the study. The behaviour of rats was recorded in a video camera and video recordings were reviewed for scoring the behaviour. It was graded as represented in Table 1 [23,24].

#### 2.6.2. Elevated Plus Maze

The elevated plus maze (EPZ) is a plus-shaped apparatus consisting of two opposing closed arms (50 cm × 10 cm × 40 cm), two opposing open arms (50 cm × 10 cm) and a central open area, elevated 50 cm above the ground. An individual rat from each group was placed in the central open area, facing one of the open arms at the beginning of the test. It could freely explore the maze for the duration of 5 min. Anxiolytics were expected to increase the proportion of entries into and time spent in open arms [25].

The whole experiment was set up in dim light conditions and without any human disturbance. Rats were pretreated with vehicle in the control group and test drugs in the drug treated groups orally 45 min before alcohol withdrawal. The test was performed at 2 h and 6 h post withdrawal period. Rats from each group were placed in central area of maze. They were left in the instrument for 5 min. Number of entries and time spent in each arm was recorded for 5 min. The behaviour of each rat was recorded in a video camera. The whole apparatus was wiped with 70% ethanol before placing the next rat in the maze. Video recording of each rat was later be analysed for the preference of the animal for the first entry, the number of entries into the open and closed arms.

#### 2.6.3. Open Field Test

Rats pre-treated orally with vehicle and test drugs 45 min before alcohol withdrawal were tested for locomotor activity in Open field apparatus. At 2 h and 6 h post withdrawal period, rats were placed in open field apparatus and were observed for 5 min. The dimensions of the box are 14.2 inch length and breadth which was divided equally into 4 squares. An increase in central locomotion or number of crossings of the lines marked was interpreted as an anxiolytic-like effect while the contrary, that is, a decrease of these variables, was associated with anxiogenic effects [26]. Number of lines crossed by rats in various treatment groups during this period were recorded.

#### 2.6.4. Pentylenetetrazol Kindling

Pentylenetetrazole (PTZ) is GABA and Glycine antagonist which is being used to induce seizures in rats. Alcohol withdrawal may lead to convulsions or make animal predisposed to convulsion. Rats entering into convulsions showed convulsive waves axially through the body, Myoclonic jerks and rearing, Clonic forelimb convulsions, Generalized tonic-clonic seizure [25]. Six hours after ethanol withdrawal, seizure threshold was measured in all the groups. Rats were placed under bell jar after administering them with PTZ with a subconvulsive dose of 30 mg/kg intraperitoneally. The behaviour of rats was observed for 30 min for onset and duration of convulsions. Number of rats which showed convulsions and percentage of rats convulsed were calculated.

### 2.7. Statistical Analysis

All raw data obtained were preprocessed to check the normal distribution and assumptions of Analysis of Variance prior the analysis. All statistical analysis was done using R version 4.0.2. Variability in the agitation score, stereotypic behaviour, open arm entries and lines crossed for the two time periods were compared using One-way ANOVA with treatment groups as the factor, followed with Tukey’s pos-hoc analysis with 95% level of significance. Weight change in the test subjects between different weeks of observations were derived and compared using a one-way ANOVA with treatment groups as factor, followed with Tukey’s post-hoc analysis at 95% level of significance. A cross-tabulation study with chi-square test at 95% level of significance was conducted to compare the frequency of convulsion recorded among the test subjects under different treatment groups.

## 3. Results

Agitation score, stereotypic behaviour score, elevated plus maze, open field test and PTZ kindling were observed and compared between the treatment groups and non-treatment group at 2nd and 6th hours of alcohol withdrawal.

### 3.1. Body Weight

It was observed that the change in body weight in all the groups was marginal. There was no significant change in the body weight of each rat which ensured that the modified liquid diet was well substituted their normal feed and ethanol consumption were uniform.

### 3.2. Behavioral Changes during Ethanol Withdrawal

#### 3.2.1. Agitation Score

**Figure 3:**
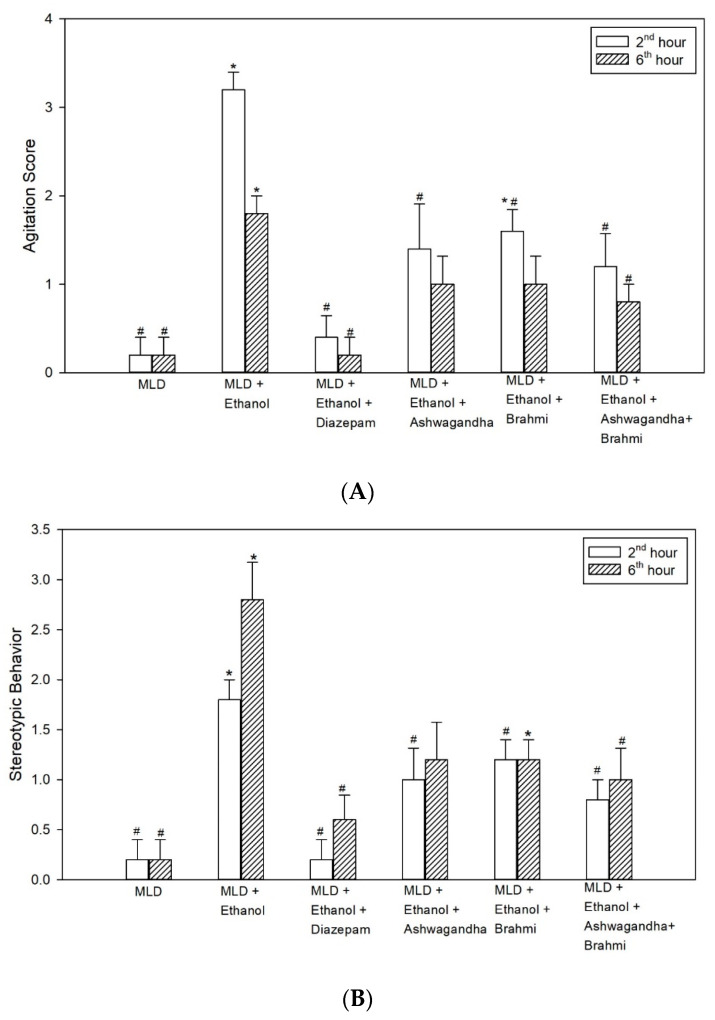
Effect of test drugs on behavioural changes (**A**) Agitation (**B**) Stereotypic behaviour at 2nd hour and 6th hour of alcohol withdrawal in rats. (‘*’ represents statistically significant compared with MLD group. ‘#’ represents statistically significance compared with MLD + Ethanol group.).

Higher agitation score in MLD + Ethanol group was observed at 2nd hour and 6th hour of alcohol withdrawal. The increase in agitation score when compared with MLD group was statistically significant at 2nd and 6th hours of alcohol withdrawal (*p* < 0.001). When compared with MLD + Ethanol group, low agitation scores observed in diazepam were statistically significant both at 2nd and 6th hours (*p* < 0.001). The lower agitation score in Ashwagandha group, when compared with MLD + Ethanol group, was statistically significant at 2nd hour (*p* = 0.006) while it was not statistically significant at 6th hour. Similarly, mean agitation score in Brahmi treated group were low at 2nd and 6th hours of alcohol withdrawal and when compared to MLD + Ethanol group, the low agitation score was statistically significant at 2nd hour (*p* = 0.046) but not at 6th hour. In the group administered with Ashwagandha + Brahmi, mean agitation score was significantly low (*p* = 0.002) at 2nd hour and 6th hour (*p* = 0.025) when compared with MLD + Ethanol (Figure 3A).

#### 3.2.2. Stereotypic Behaviour

The higher stereotypic behaviour score observed in the untreated ethanol group when compared with MLD group was statistically significant at 2nd and 6th hours of alcohol withdrawal (*p* < 0.001). Mean stereotypic behaviour score in diazepam treated group when compared with MLD + Ethanol group significantly reduced both at 2nd and 6th hours of alcohol withdrawal (*p* < 0.001). Reduced stereotypic behaviour score in Ashwagandha group, when compared with MLD + Ethanol group, was statistically significant at 2nd hour (*p* = 0.009) while it was not statistically significant at 6th hour. Similarly, mean stereotypic behaviour score in Brahmi treated group when compared to MLD + Ethanol group was significantly lower at 2nd hour (*p* = 0.009) and not at 6th hour. In the group administered with Ashwagandha + Brahmi stereotypic behaviour score was significantly low when compared with MLD + Ethanol group at 2nd hour (*p* = 0.003) and 6th hour (*p* = 0.043) (Figure 3B). Representative video recording of control rat is provided as Supplementary Material (Video S1 Grooming).

### 3.3. Elevated Plus Maze (EPZ)

**Figure 4:**
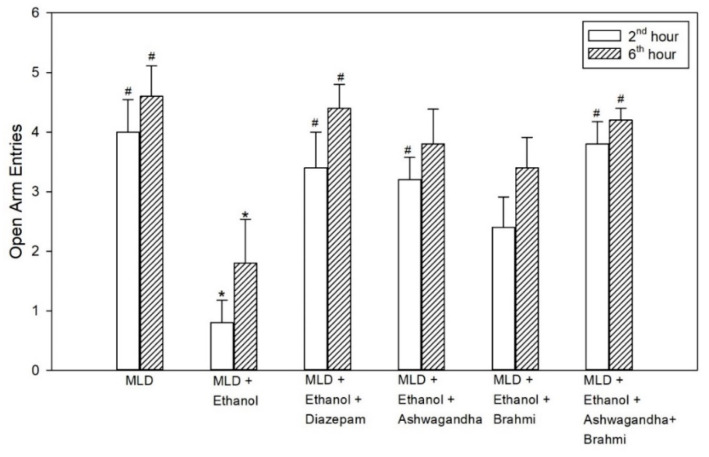
Effect of test drugs on anxiety represented by the number of open arm entries in elevated maze test at 2nd hour and 6th hour of alcohol withdrawal in rats. (‘*’ represents statistically significant compared with MLD group. ‘#’ represents statistically significance compared with MLD + Ethanol group.).

The decrease in open arm entries in the untreated ethanol group was statistically significant when compared with the MLD group (*p* values = 0.001 and *p* = 0.009) and in Diazepam treated group, the increase in open arm entries compared with the untreated ethanol group was found to be statistically significant (*p* = 0.008 and *p* = 0.018) at 2nd hour and 6th hour of alcohol withdrawal. However, the increase in open arm entries of the Ashwagandha group was statistically significant at 2nd hour (*p* = 0.016) and not at 6th hour from the untreated ethanol group. In the group administered with Brahmi, mean open arm entries were not statistically significant from the untreated ethanol group. Similarly, in Ashwagandha + Brahmi treated group, mean open arm entries at 2nd and 6th hour of alcohol withdrawal were statistically significant from the untreated ethanol group (*p* = 0.002 and *p* = 0.033) (Figure 4). The first entry of rats into an open arm or closed arm of EPZ at 2nd and 6th hour of alcohol withdrawal of all the groups was tabulated in Table 2. Representative video recording of Ashwagandha + Brahmi treated rat is provided as Supplementary Material (Video S2 Elevated Plus Maze).

### 3.4. Open Field Test

**Figure 5:**
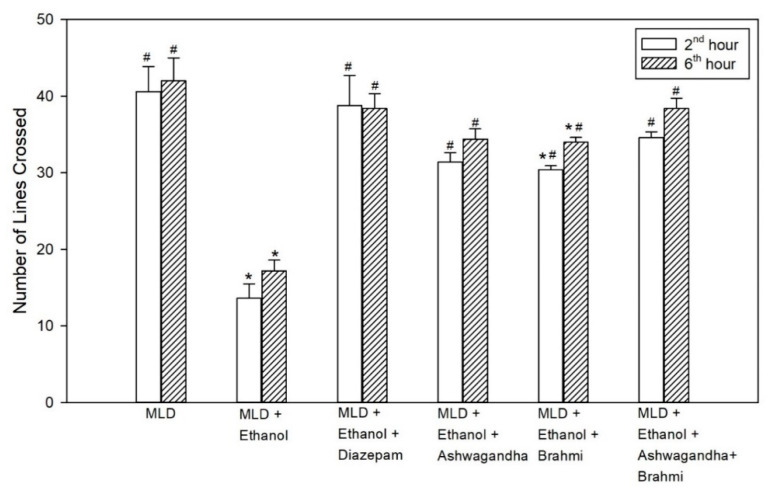
Effect of test drugs on locomotor activity represented by the number of lines crossed in open field test at 2nd hour and 6th hour of alcohol withdrawal. (‘*’ represents statistically significant compared with MLD group. ‘#’ represents statistically significance compared with MLD + Ethanol group).

The decrease in locomotor activity in the untreated ethanol group compared to MLD group was statistically significant (*p* < 0.001). The increase in locomotor activity observed in the diazepam treated group when compared to the untreated ethanol group was statistically significant both at 2nd and 6th hour (*p* < 0.001). In the group administered with Ashwagandha, the increase in the number of lines crossed compared to the untreated ethanol group was statistically significant (<0.001). Similarly, in the Brahmi treated group, there was a statistically significant increase in the mean number of lines crossed compared to the untreated ethanol group at 2nd and 6th hour of alcohol withdrawal (*p* < 0.001). In Ashwagandha + Brahmi treated group, mean number of lines crossed in the open field were significantly different from MLD + Ethanol group (*p* < 0.001) (Figure 5). Representative video recording of control rat is provided as Supplementary Material (Video S3 Exploratory Behavior).

### 3.5. Pentylenetetrazol (PTZ) Kindling

**Figure 6:**
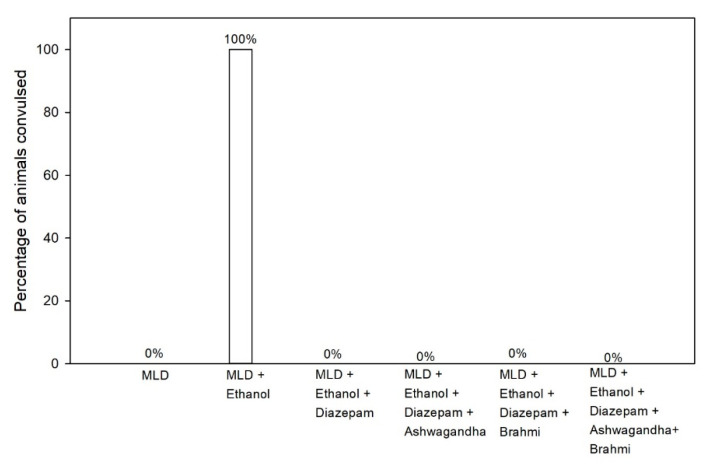
Effect of test drugs on seizures activity represented by the percentage of animals convulsed at 6th hour of alcohol withdrawal after Pentylenetetrazol kindling.

In MLD group, administration of sub-convulsive dose of PTZ (30 mg/kg i.p) has not shown any convulsions (0% convulsion), whereas the same dose of PTZ administration in MLD + Ethanol group at 6th hour of alcohol withdrawal resulted in the convulsion of all rats (100% convulsion). In groups treated with diazepam, Ashwagandha, Brahmi and Ashwagandha + Brahmi, no rats developed any convulsion on PTZ administration (30 mg/kg i.p) at 6th hour of alcohol withdrawal (0% convulsion) (Figure 6). Representative video recording of MLD + Ethanol treated rat is provided as Supplementary Material (Video S4 PTZ kindling).

## 4. Discussion

Alcohol abuse is a major health problem in our society. One of the features of chronic alcohol intake is the development of physical dependence, resulting in physical withdrawal reactions after cessation of alcohol. Alcohol withdrawal includes symptoms such as neural excitation (seizures), disorientation, anxiety and agitation [27].

Long-term exposure to alcohol causes adaptive changes in several neurotransmitter systems, especially gamma-aminobutyric acid (GABA) receptors and glutamate receptors. GABA plays a key role in the neurochemical mechanisms on the basis of intoxication, tolerance and withdrawal. Repeated exposure to alcohol reduces GABA hyperpolarization resulting in reduced neural inhibition [28].

Benzodiazepines like diazepam, chlordiazepoxide, etc. are the recommended drugs for the treatment of alcohol withdrawal symptoms because they ameliorate both the anxiety and seizures. They depress CNS excitation by GABA agonism. However, they have potential of causing dependence and leads to abuse. Benzodiazepines also possess serious adverse effects like respiratory failure and delirium [29,30].

Ashwagandha and Brahmi are commonly studied herbs in various CNS disorders including anxiety and seizures [31]. Both of these have shown significant GABA agonist activity in few preclinical studies [16,32]. In our study, we observed an improvement in the behavioural activity of rats with alcohol withdrawal in diazepam, Ashwagandha, Brahmi and Ashwagandha + Brahmi treated groups.

In elevated plus maze (EPM), we observed an anxiolytic effect in alcohol withdrawal rats when treated with Ashwagandha, Brahmi, Ashwagandha + Brahmi and diazepam. The administration of test drugs resulted in first entry of majority of rats into the open arm. In addition, the number of entries into open arms were higher in Ashwagandha, Brahmi and Ashwagandha + Brahmi groups compared with the untreated ethanol group. However, none of these anxiolytic effects were statistically significant when analysed. Previous reports based on the EPM test suggest that withdrawal of ethanol following chronic treatment generates anxiety which is a measure of psychological dependence [33,34]. The present studies confirm these by showing a significant reduction in open arm entries in the MLD + Ethanol group at 2nd and 6th hours of alcohol withdrawal. In addition, in a previous study, Ashwagandha showed a significant anxiolytic effect in alcohol withdrawal rats in EPM at 100, 200 and 500 mg/kg oral dose [10].

In the open field test, alcohol withdrawal resulted in reduced locomotor activity at the 2nd and 6th hours. The reduction in exploratory behaviour, often referred to as withdrawal anxiety, was observed by the decrease in the number of line crossing activity. When treated with diazepam, rats showed a significant increase in the number of line crossings. Rats treated with Ashwagandha, Brahmi and Ashwagandha + Brahmi also showed an increase in locomotor activity. In a previous study, Ashwagandha (500 mg/kg, oral) showed a significant increase in locomotor activity in the open field at 6th hour of alcohol withdrawal [35]. Mishra et al. also reported that rats treated with Ashwagandha (3.75 mg of withanolide glycosides per kg body weight) and Brahmi (10 mg of bacosides per kg body weight) were effective in controlling agitation and seizures and showed similar results [36].

Previous studies showed a persistent reduction in the seizure threshold in rats on chronic ethanol administration [35,37]. In this study, Ashwagandha, Brahmi and their combination have suppressed PTZ kindling seizures in alcohol withdrawal rats similar to diazepam. In the previous study, Aswagandha at higher dose (500 mg/kg) suppressed seizures in a similar manner, whereas Ashwagandha at lower dose (200 mg/kg) did not suppress seizures at same level [35]. The findings in our study show that both Ashwagandha and Brahmi can have a similar GABA agonist activity comparable to benzodiazepines which offers significant protection against convulsions in alcohol withdrawal syndrome.

## 5. Conclusions

Alcohol withdrawal syndrome is a common manifestation in alcohol dependence due to the imbalance of GABA-NMDA homeostasis in CNS caused by chronic alcohol intake. Similar to diazepam, Ashwagandha and Brahmi individually and in combination significantly reduced the behavioural activity, PTZ kindling seizures and increased the locomotor activity in alcohol withdrawal rats. Further studies at different doses of Ashwagandha and Brahmi can be explored to evaluate their association with alcohol withdrawal syndrome.

## Figures and Tables

**Table 1:** Scoring of agitation and stereotyped behaviour.

Signs	Scoring	Characteristics Observed
Agitation	0	No irritability or aggressive behaviour
	1	Rats showing mild or moderate irritability
	2	Very irritable
	3	handling vocalization and moderately aggressive
	4	handling vocalization and very aggressive
	5	spontaneous vocalization and very aggressive
Stereotyped behaviour	0	no stereotyped behaviour
	1	rats showing only one stereotyped behaviour
	2	two stereotyped behaviour
	3	three stereotyped behaviour
	4	four stereotyped behaviour
	5	all stereotyped behaviour

**Table 2:** Elevated plus maze first entry in different groups at 2nd and 6th hour of alcohol withdrawal.

Groups	First Entry (Percentage of Rats)			
	2 h		6 h	
	Open Arm	Closed Arm	Open Arm	Closed Arm
MLD	80%	20%	80%	20%
MLD + Ethanol	0%	100%	20%	80%
MLD + Ethanol + Diazepam	60%	40%	80%	20%
MLD + Ethanol + Ashwagandha	60%	40%	60%	40%
MLD + Ethanol + Brahmi	40%	60%	60%	40%
MLD + Ethanol + Ashwagandha + Brahmi	60%	40%	60%	40%

MLD—Modified liquid diet.

## Data Availability

The datasets used and/or analysed during the current study are available from the corresponding author on reasonable request.

## Supplementary Materials

The following are available online at https://www.mdpi.com/article/10.3390/brainsci11070919/s1, Video S1 Grooming; Video S2 Elevated Plus Maze; Video S3 Exploratory Behavior; Video S4 PTZ kindling
